# Sex Differences under Vitamin D Supplementation in an Animal Model of Progressive Multiple Sclerosis

**DOI:** 10.3390/nu16040554

**Published:** 2024-02-17

**Authors:** Michaela Tanja Haindl, Muammer Üçal, Cansu Tafrali, Willibald Wonisch, Cigdem Erdogan, Marta Nowakowska, Milena Z. Adzemovic, Christian Enzinger, Michael Khalil, Sonja Hochmeister

**Affiliations:** 1 Department of Neurology, Medical University of Graz, 8010 Graz, Austria; 2 Department of Neurosurgery, Medical University of Graz, 8010 Graz, Austria; 3 Otto Loewi Research Center, Department of Physiological Medicine, Medical University of Graz, 8010 Graz, Austria; 4 Department of Clinical Neuroscience, Karolinska Institutet, 171 77 Stockholm, Sweden

**Keywords:** vitamin D, sex-associated differences, progressive multiple sclerosis

## Abstract

A central role for vitamin D (VD) in immune modulation has recently been recognized linking VD insufficiency to autoimmune disorders that commonly exhibit sex-associated differences. Similar to other autoimmune diseases, there is a higher incidence of multiple sclerosis (MS) in women, but a poorer prognosis in men, often characterized by a more rapid progression. Although sex hormones are most likely involved, this phenomenon is still poorly understood. Oxidative stress, modulated by VD serum levels as well as sex hormones, may act as a contributing factor to demyelination and axonal damage in both MS and the corresponding preclinical models. In this study, we analyzed sex-associated differences and VD effects utilizing an animal model that recapitulates histopathological features of the progressive MS phase (PMS). In contrast to relapsing–remitting MS (RRMS), PMS has been poorly investigated in this context. Male (*n* = 50) and female (*n* = 46) Dark Agouti rats received either VD (400 IU per week; VD^+^) or standard rodent food without extra VD (VD^−^) from weaning onwards. Myelination, microglial activation, apoptotic cell death and neuronal viability were assessed using immunohistochemical markers in brain tissue. Additionally, we also used two different histological markers against oxidized lipids along with colorimetric methods to measure protective polyphenols (PP) and total antioxidative capacity (TAC) in serum. Neurofilament light chain serum levels (sNfL) were analyzed using single-molecule array (SIMOA) analysis. We found significant differences between female and male animals. Female rats exhibited a better TAC and higher amounts of PP. Additionally, females showed higher myelin preservation, lower microglial activation and better neuronal survival while showing more apoptotic cells than male rats. We even found a delay in reaching the peak of the disease in females. Overall, both sexes benefitted from VD supplementation, represented by significantly less cortical, neuroaxonal and oxidative damage. Unexpectedly, male rats had an even higher overall benefit, most likely due to differences in oxidative capacity and defense systems.

## 1. Introduction

Multiple sclerosis (MS) is a chronic inflammatory demyelinating disease of the central nervous system caused by an autoimmune attack on myelin sheaths [1]. Despite widespread research efforts, some pathomechanisms behind this disease remain unclear. Even though current immunomodulatory medications improved MS treatment by reducing relapses, they often fail to stop disease progression, underlining the need for developing new therapeutic options. Over the last few years, a central role for vitamin D (1,25-dihydroxyvitamin D; VD) in immune modulation has been recognized, linking VD insufficiency to autoimmune disorders that commonly display significant differences between females and males due to genetic, epigenetic, hormonal and environmental factors. Like other autoimmune diseases, females are more susceptible than males to developing MS, but men accumulate disability more rapidly than women [2]. However, the findings on faster disease progression in male MS patients remain controversial and there is no clear mechanism that explains the quicker progression in men but the higher disease incidence in women yet [1]. Most likely, sex hormones differently affect the immune system. In general, males show a slight immunosuppression compared to females, maybe due to androgens, while females show a higher immunoreactivity, likely related to estrogens. This leads to a greater resistance to infections but also to a higher risk of developing autoimmune diseases [3,4]. There is accumulating evidence indicating a crosstalk between VD and the sex hormone estrogen, indicating a further layer of complexity [5,6,7]. Thus, the assessment of the therapeutic or protective effects of VD in autoimmune diseases would be inadequate unless sex differences are properly addressed [3,4]. Furthermore VD has been shown to exert protective effects in oxidative stress, which is discussed as a mediator of demyelination and axonal damage in both MS and animal models [8,9].

Currently, there is an abundance of research available on the use of VD supplementation, sex differences and the role of oxidative stress in the relapsing–remitting form of MS (RRMS), but not in the progressive disease phase of MS (PMS). Only an association between low VD status and early conversion to the secondary progressive MS phase has been assumed, and a role of VD in myelin protection has been discussed [10,11]. These findings suggest a protective effect of VD in PMS. Nevertheless, the mechanism of such a potentially beneficial influence, the effect of sex-based differences and finally whether and how VD supplementation could influence the disease course in PMS remains unclear.

Another issue is related to the heterogeneity of the disease. Therefore, basic research is a valuable and essential tool to achieve standardized conditions. Research into preclinical models so far has primarily focused on the cellular hallmarks of RRMS, slightly indicating males having a greater progression and higher mortality due to specific astrocytic factors and iron accumulation [2]. To overcome the lack of suitable animal models for PMS, our research team developed an animal model recapitulating the cellular features of the progressive disease phase well [12]. In the present study, we used this model to gain more insight into the effect of VD on the cellular hallmarks of PMS and analyze sex-based differences between animal groups.

## 2. Materials and Methods

### 2.1. Animals

In total, 96 Dark Agouti (DA) rats (46 female, 50 male) aged 10–12 weeks, obtained from Janvier, France, underwent the experimental procedure described in detail in [12]. Rats with an implanted catheter only served as healthy-appearing controls (HAC; female = FHAC, *n* = 7; male = MHAC, *n* = 9). A detailed list of used animals and group names is given in Table 1. A list of all abbreviations is given in Supplementary Table S1. All animal experiments were carried out under the approval of the local authorities (Federal Ministry of Science and Research, Vienna, Austria; 66.010/0072-WF/V/3b/2017, 10.12.2019).

### 2.2. Experimental Setup

**Figure 1:**
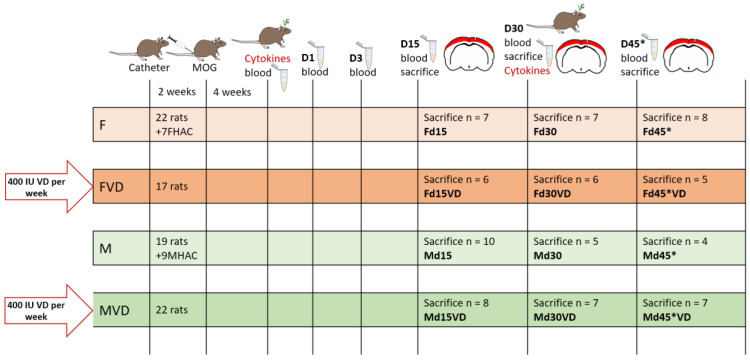
Experimental setup. There were four experimental groups, split into female VD^−^ (F), female VD^+^ (FVD), male VD^−^ (M) and male VD^+^ (MVD) rats. Additionally, 7 female healthy-appearing controls (FHAC) and 9 male-healthy appearing controls (MHAC) were used. VD^+^ rats received 400 IU VD per week from weaning until the day of sacrifice. The experiment started with the catheter implantation, and after a healing period of 2 weeks, animals were immunized with MOG and the antibody titer was checked 4 weeks later. The blood–brain-barrier was opened via the injection of a proinflammatory cytokine mixture and the first signs of cortical pathology start on d1. The timeline indicates blood sampling (“blood”), days of sacrifice (“sacrifice”) and points of cytokine injection (“cytokines”). Corresponding animal numbers are given for the sacrifices on d15, d30 and d45* for the four different groups. The asterisk at d45 indicates an additional cytokine injection on d30 to initiate a second disease episode.

The experimental setup is described in detail elsewhere [12]. Briefly, cortical demyelination was induced by the delivery of pro-inflammatory cytokines through an implanted catheter in myelin oligodendrocyte glycoprotein (MOG)-pre-immunized rats. The VD-supplemented animal groups (VD^+^) received 1 additional drop of VD (=400 IU Vitamin D; Fresenius-Kabi, Graz, Austria) per week starting from weaning (at age 3 weeks) until the day of sacrifice. VD was administered via a Pasteur pipette by hand directly into the rats’ mouth to ensure that the animals received the desired dosage. All other groups received standard rodent food only (VD^−^). The peak of cortical pathology in this model can be observed on day (d) 15. On d30, the first cortical remyelination is detectable and even a second disease phase or relapse can be generated by a second cytokine injection on d30, indicated as d45*. An overview of the experimental setup is given in Figure 1.

### 2.3. Blood Withdrawal, Euthanasia and Tissue Harvesting

Peripheral blood was collected via tail tip snip before catheter implantation (FHAC, MHAC), after MOG immunization (FMOG, MMOG) and on d1, d3, d15, d30 and d45* after cytokine injection. Serum was harvested by means of centrifugation at 2000 g twice for 10 min, one hour after blood sampling, and immediately stored at −70 °C until use according to the guidelines [13]. Animals were sacrificed on d15, d30 and d45*: deep anesthesia was induced with 4% isoflurane followed by intraperitoneal injection of 25 mg thiopental (Sandoz, Kundl, Austria) and transcardial perfusion with 4% paraformaldehyde (PFA; Merck, Darmstadt, Germany) in phosphate-buffered saline (PBS, pH = 7.4). Brains and spinal cords were dissected and post-fixed in 4% PFA for 24 h. Only brains were used for further detailed histopathological analysis, as the spinal cords are unaffected in this animal model and are only routinely analyzed for potential affection [12].

### 2.4. Histopathology and Immunohistochemistry

The tissue was sectioned into 1.5 µm pieces and fixed in paraffin. Sections were dewaxed in xylol (Fisher Thermo Scientific, Schwerte, Germany) for immunohistochemical (IHC) staining, rehydrated, and steamed in citric acid for one hour [12,14]. Following a 20 min room temperature incubation period with 2.5% normal horse serum (Vector Laboratories, Burlingame, CA, USA), slices were coated with primary antibodies (Supplementary Table S2) and incubated at 4 °C overnight. For all IHC investigations, the ImmPRESS System (Vector Lab¸ secondary antibodies) was utilized, visualized with 3,3′diaminobenzidine-tetrahydrochloride (DAB, Sigma-Aldrich, Buchs, Switzerland) and counterstained with hematoxylin (all IHC analyses, except for the caspase 3 labeling). After dehydration, slides were covered with Shandon Consul-Mount (Fisher Thermo Scientific) and a coverslip.

### 2.5. Quantitative Histopathological Evaluation

The quantification of cellular markers for demyelination (proteolipid protein; PLP), microglial activation (ionized calcium-binding adaptor molecule 1; Iba1), apoptotic cells (caspase 3; Casp3), neuronal cell loss (neuronal nuclei; NeuN) and oxidative stress (Cu++ oxidized LDL and HOCl oxidized LDL; Cu++oxLDL and HOCloxLDL) was performed by one investigator blinded for experimental groups. For this purpose, positively stained cells (Iba1, Casp3, NeuN, Cu++oxLDL and HOCloxLDL) were counted in three full optical grids on the ipsilateral (ipsi; catheter area) and contralateral side (contra; at the corresponding contralateral area) using a 20x objective lens of the microscope (Zeiss AXIO Imager.M2). The average value was converted into the number of immunoreactive (“positive”) cells per mm^2^. Demyelination was assessed via the quantification of the loss of PLP immunoreactivity in single squares of the full optical grid covering the ipsilateral or contralateral side, respectively, using a 20x objective lens. Values were then transformed into the loss of PLP immunoreactivity per mm^2^.

### 2.6. Colorimetric Methods for Oxidative Capacity and Polyphenols

To evaluate differences in the antioxidative effects between male and female rats, we used two different test systems, i.e., the total antioxidative capacity (TAC^®^; Omnignostica Ltd., Klosterneuburg, Austria) and polyphenols (PPm^®^; Omnignostica Ltd., Höflein, Austria), as described previously [15]. The TAC assay was performed according to the manufacturer’s instructions with distinct modifications for measuring small sample volumes [16]. No modifications were required for the PP test. Note that the TAC assay is sensible to hemolytic samples, which is why the sample numbers were reduced compared to the more robust PP test.

### 2.7. NfL Measurement by Single-Molecule Array (SIMOA)

Serum NfL was measured using a commercial Simoa^®^ NF-light™ Advantage Kit on a HDX analyzer (Quanterix, Billerica, MA, USA) based on single-molecule arrays and the simultaneous counting of single captured microscopic beads carrying antibody complexes. The analytical sensitivity of this technique enables a reliable measurement of low NfL concentrations in blood samples [17]. Advanced SIMOA NfL-kits (Quanterix, MA, USA) developed for human research use were utilized according to the manufacturer’s instructions, since there is a high cross-reactivity for rats.

### 2.8. Statistical Analysis

Statistical analysis was performed using SPSS Statistics (v23, IBM, Armonk, NY, USA), and graphs were illustrated in Prism (v9, GraphPad Software, La Jolla, CA, USA). A detailed list of statistical methods used per experiment and corresponding results is given in Table 2 for histology and in Table 3 for results of serum biomarkers. The text body and all graphs provide median values and a 95% confidence interval (CI). A difference of *p* < 0.05 was considered statistically significant.

## 3. Results

### 3.1. Histological Analysis Revealed Benefits of VD Supplementation and General Sex-Differences

**Figure 2:**
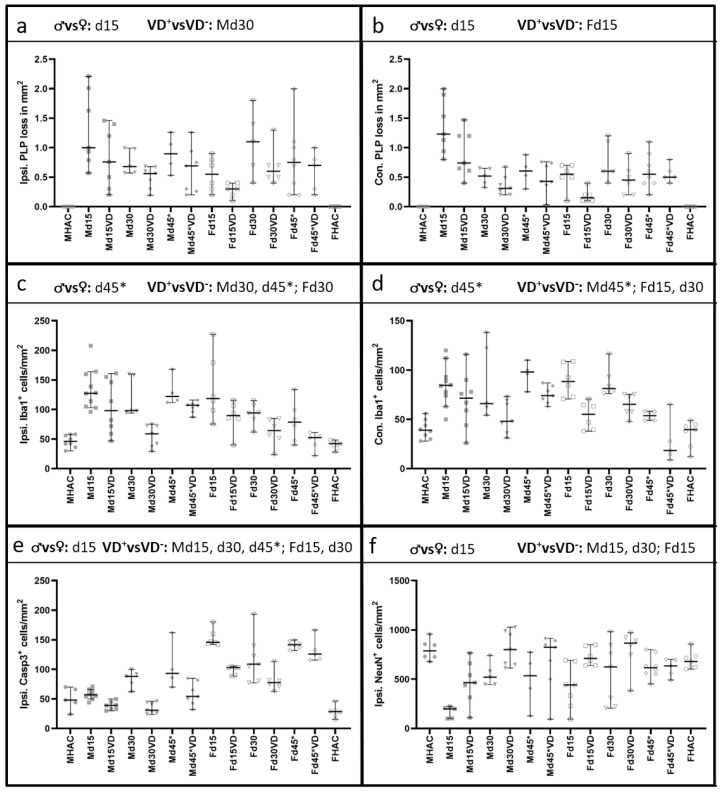
Quantification of IHC staining. Significant comparisons are indicated in the heading of each graph. Original data points are shown with different symbols, and the median and 95% CI are represented in each graph. PLP loss is displayed in the first line in (**a**) on the ipsi and in (**b**) on the contra side. Male animals show a disease peak on d15, with a PLP loss of 1.00 [95% CI = 0.57–2.21] per mm^2^. Overall, VD^+^ male animals benefit from supplementation, reaching statistical significance only on d30 (*p* = 0.050). A similar pattern can be observed on the contra side in male rats but without statistical significance. Female rats show a different pattern, with the most pronounced cortical demyelination on d30 of 0.55 [95% CI = 0.20–0.90] per mm^2^. Also, female animals benefit from VD supplementation, but statistical significance is only reached on the contra side on d15 (*p* = 0.027), with a similar course as seen on the ipsi side. Males again show a similar pattern regarding microglial activation on the ipsi (**c**) and the contra (**d**) side. There are significantly fewer Iba1-positive cells in VD^+^ male animals on d30 and 45* on the ipsi side (*p* = 0.004; *p* = 0.018) and on d45* on the contra side (*p* = 0.038). On the ipsi side, female rats show the highest microglial activation on d15 on both sides, with a significant lower microglial activation in VD^+^ females on d15 (contra *p* = 0.005) and d30 (ipsi *p* = 0.025; contra *p* = 0.004). Ipsilateral Casp3 quantification is shown in (**e**), with all VD^+^ groups revealing significantly fewer apoptotic cells except for Fd45*. VD supplementation has a significant effect on neuronal survival (**f**) in Md15 (*p* = 0.019), Md30 (*p* = 0.019) and Fd15 (*p* = 0.022). Differences between the sexes are detectable on d15 for PLP ipsi (*p* = 0.022), contra (*p* = 0.003), Casp3 (*p* = 0.001) and NeuN (*p* = 0.042). Microglial activation shows a significant difference between the sexes on d45* on both sides with *p* values of 0.050 (ispi.) and 0.010 (contra).

**Figure 3:**
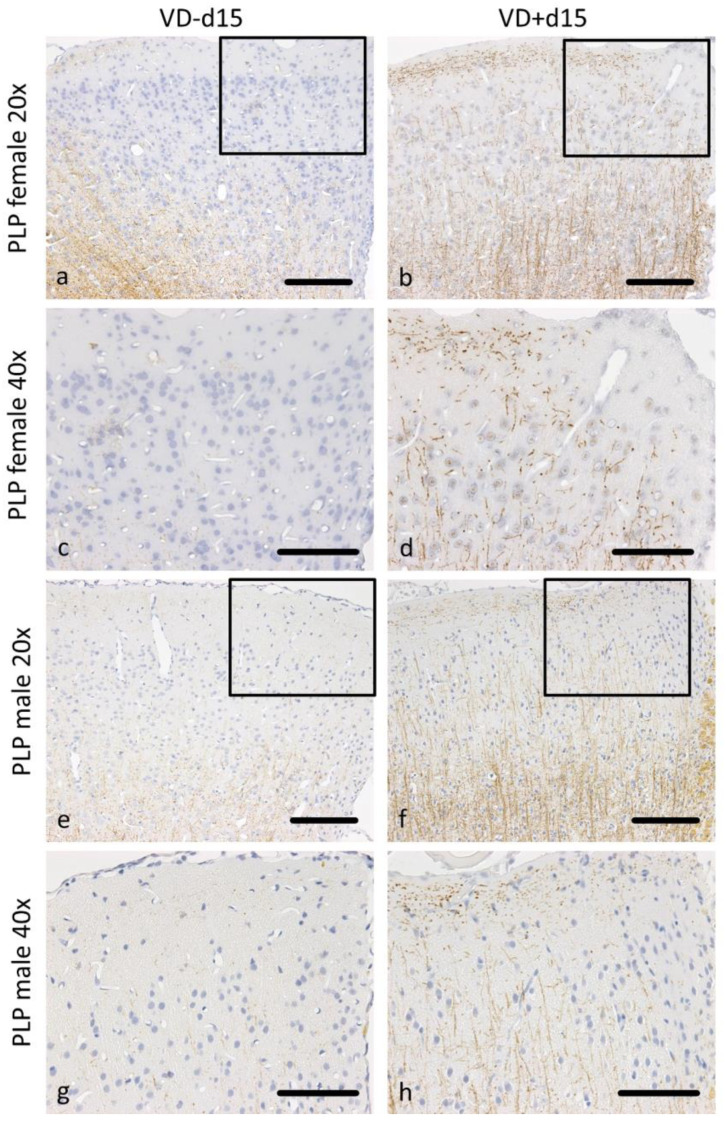
Representative microscopic pictures of PLP staining on d15. All pictures represent the upper right corner of the catheter puncture. The black rectangle in the 20× pictures marks the area shown in the next row at a higher magnification (40×). In female rats, cortical demyelination can be observed in (**a**,**c**), with no remaining signal of PLP positive fibers. In comparison, VD^+^ supplemented females show traces of PLP-positive areas (**b**,**d**). A similar pattern can be observed in male animals, with wide areas of cortical demyelination observable in (**e**,**g**) and PLP preservation in (**f**,**h**). Scale bars represent 100 µm.

Figure 2 illustrates the quantification of PLP loss for the (a) ipsi and (b) contra sides. Figure 3 shows corresponding representative microscopy pictures. We observe that VD^+^ animals generally exhibit better PLP preservation (Figure 2a,b; Figure 3b,d,f,h), which was reflected in the brain tissue from males on d30 ipsi (*p* = 0.050) as well as in females on d15 contra (*p* = 0.027) compared to the VD^−^ groups. Moreover, there was a significant detectable difference between males and females (VD^−^) on d15 on both sides (ipsi *p* = 0.022; contra *p* = 0.003).

**Figure 4:**
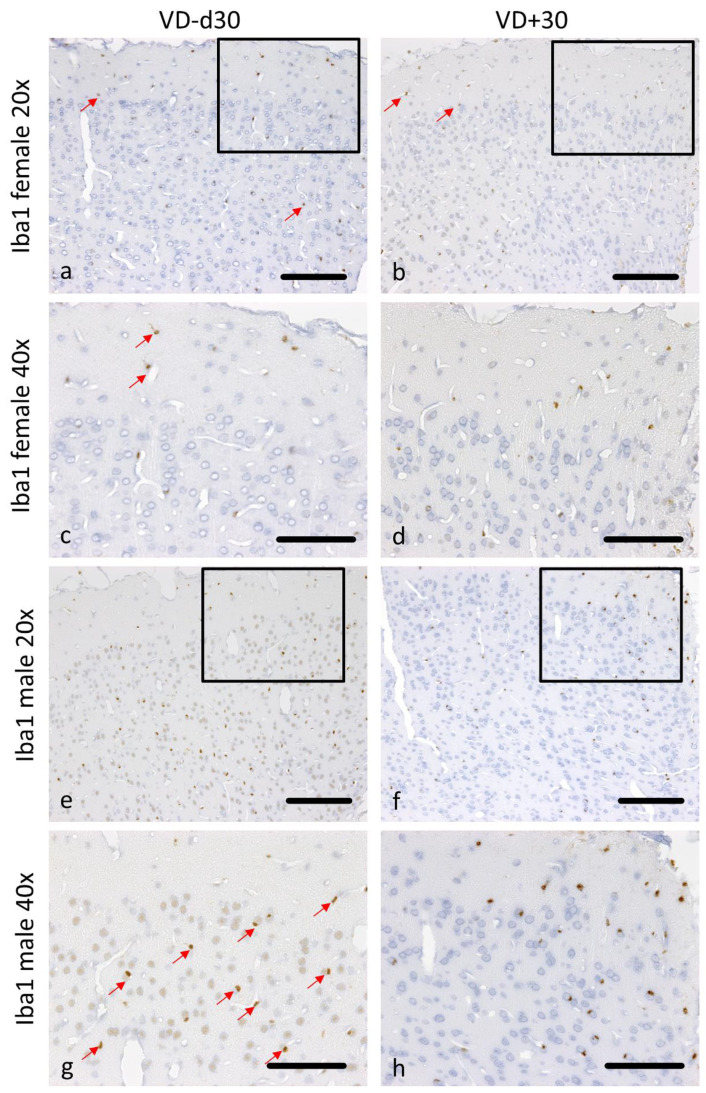
Representative microscopic pictures of Iba1 staining on d30. All pictures represent the upper right corner of the catheter puncture. The black rectangle in the 20× pictures marks the area shown in the next row at a higher magnification (40×). Red arrows point at positively stained Iba1 cells. In comparison to VD^−^ animals (**a**,**c**), less microglial activation is detectable in female VD^+^ animals (**b**,**d**). The same pattern can be observed in male rats (**e**–**h**). Scale bars represent 100 µm.

We further observed significantly less pronounced microglial activation (Figure 2c,d) in VD^+^ male animals on d30 (ipsi *p* = 0.004) and d45* (ipsi *p* = 0.018 and contra *p* = 0.038). Females have shown a different pattern with significantly less microglial activation in VD^+^ animals on d15 (contra *p* = 0.005) and d30 (ipsi *p* = 0.025 and contra *p* = 0.004). Additionally, there was a significant difference between males and females (VD^−^) on d45* on both hemispheres (ipsi *p* = 0.050; contra *p* = 0.010). Figure 4a–h shows corresponding representative microscopic pictures of the microglial activation of all groups.

**Figure 5:**
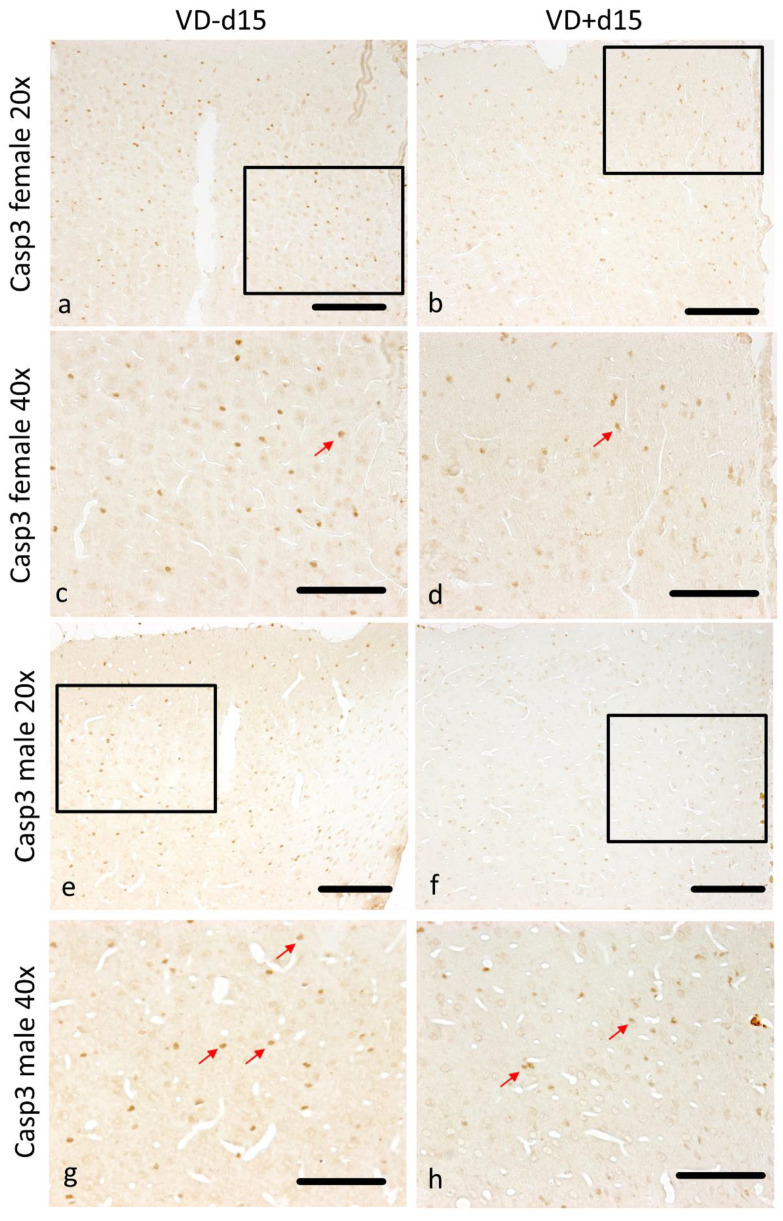
Representative microscopic pictures of Casp3 staining on d15. All pictures represent the upper right corner of the catheter puncture. The black rectangle in the 20× pictures marks the area shown in the next row at a higher magnification (40×). Red arrows point at positively stained Casp3 cells. Overall, there are many more Casp3-positive cells detectable in females (**a**–**d**) than in males (**e**–**h**). Nevertheless, both sexes benefit from VD supplementation, with fewer apoptotic cells detectable (**b**,**d**,**f**,**h**). Scale bars represent 100 µm.

The quantification of Casp3 staining revealed a benefit from VD in all groups except for Fd45* (Figure 2e), representing significantly fewer apoptotic cells on the ipsi side (Md15 *p* = 0.002, Md30 *p* = 0.004, Md45* *p* = 0.023, Fd15 *p* = 0.003, Fd30 *p* = 0.046). This pattern is also represented in Figure 5a–h. There are significantly more apoptotic cells in female rats (VD^−^) detectable in comparison to males (VD^−^) on d15 (*p* = 0.001).

**Figure 6:**
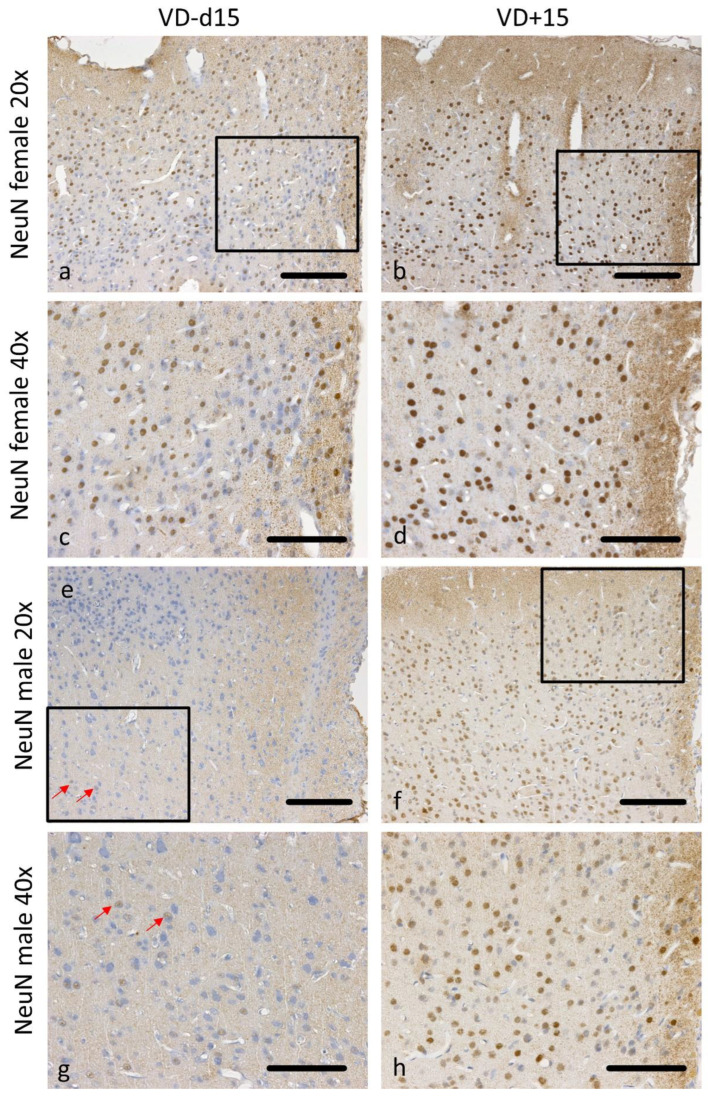
Representative microscopic pictures of NeuN staining on d15. All pictures represent the upper right corner of the catheter puncture. The black rectangle in the 20× pictures marks the area shown in the next row at a higher magnification (40×). Red arrows point at positively stained NeuN cells. Overall, there are many more neurons preserved in females (**a**–**d**) than in males (**e**–**h**). Nevertheless, both sexes benefit from VD supplementation, with a higher neuronal survival (**b**,**d**,**f**,**h**). Scale bars represent 100 µm.

Neuronal cell loss was significantly reduced in male VD^+^ rats on d15 and d30 (*p* = 0.019) and in female VD^+^ rats on d15 (*p* = 0.022). We also noted a significant sex-associated difference on d15 (VD^−^; *p* = 0.042), reflected in a better neuronal cell preservation in females (Figure 2f). Representative microscopic pictures of NeuN are shown in Figure 6a–h.

### 3.2. NfL Serum Level (sNfL) Is Lowered with VD Supplementation Only in Male VD^+^ Animals

**Figure 7:**
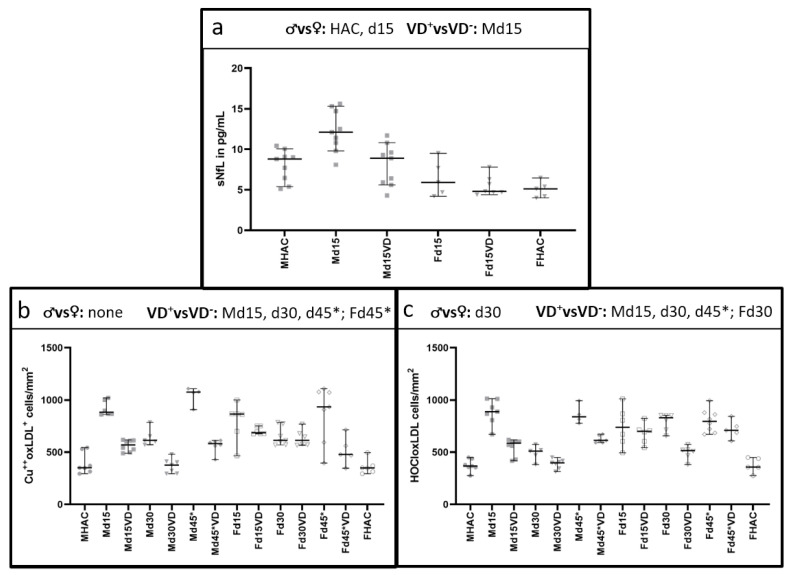
Differences in sNfL between the sexes and oxidative stress markers in histology between VD^+^, VD^−^ and the sexes. Significant comparisons are indicated in the heading of each graph. NfL serum level is significantly higher in male animals (*p* = 0.016) compared to female rats (**a**). VD supplementation leads to a significant sNfL reduction only in male animals (*p* = 0.006). With values ranging only from 4.8 [95% CI = 4.4–7.8] pg/mL to 5.9 [95% CI = 4.2–9.5] pg/mL over all female groups, there is no significant difference detectable. Both markers for oxidative stress (**b**,**c**) show a similar trend towards the male experimental groups, with a peak on d15 and d45* in male animals. In female animals, there is an additional peak on d30 for the HOCl oxLDL marker (**c**). Overall, there is a significant reduction in both oxidative stress markers in male VD^+^ animals (*p* = 0.001, *p* = 0.042, respectively). A different pattern is observed in female rats, where there is only a significant difference detectable between VD^+^ and VD^−^ rats on d30 for the HOCLoxLDL marker (**c**) and on d45* for Cu++oxLDL marker (**b**). Despite these differences between VD^+^ and VD^−^ animals in males and females, a significant difference between the sexes is only detectable on d30 (*p* = 0.006).

Serum NfL levels differed significantly between male and female rats (Figure 7a), with higher sNfL detectable in males (*p* = 0.016). While VD supplementation led to a significant reduction in sNfL in male rats on d15 (*p* = 0.006), no such difference was detectable on d15 in females (*p* = 0.683). On d15, sNfL was significantly higher in male than in female VD^−^ rats (*p* = 0.004).

### 3.3. Histological Oxidative Stress Markers Are Lower Mainly in Male VD^+^ Animals

Both markers for oxidative stress showed a similar trend to the male experimental groups (Figure 7b,c). Both markers reached their highest levels on d15 and d45* in untreated male animals, concomitant with the highest PLP loss. In females, there was an additional peak on d30 for the HOCl-oxLDL marker. Both oxidative stress markers were significantly reduced in male VD^+^ animals (*p* = 0.001–0.042; Table 3). A different pattern was observed in female rats, with only a significant difference detectable between VD^+^ and VD^−^ rats on d30 for the HOCL-oxLDL marker and on d45* for Cu++-oxLDL marker. Notably, a significant difference between the sexes was detectable on d30 (*p* = 0.006).

### 3.4. Total Antioxidant Capacity and Polyphenols Are Increased in Female Rats

**Figure 8:**
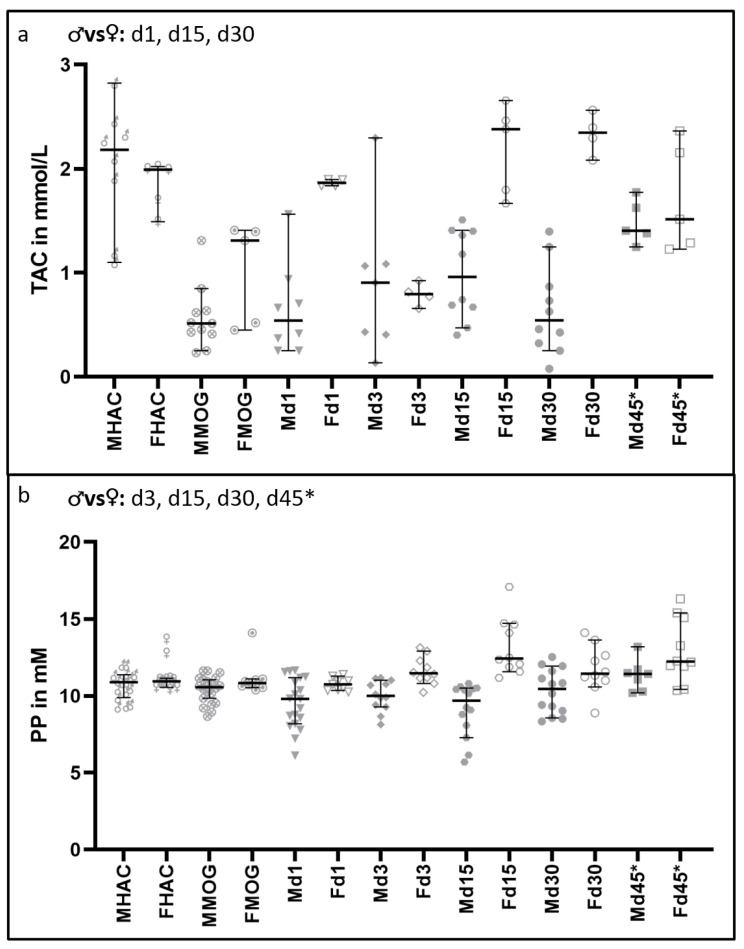
Differences in oxidative stress markers in serum between male and female rats. Significant comparisons are indicated in the heading of each graph. In (**a**), the TAC results are shown. There is a significant difference in TAC detectable after MOG immunization in comparison to HAC (FHAC to FMOG *p* = 0.025; MHAC to MMOG *p* = 0.001), but there is no significant difference detectable in that TAC decrease between the sexes (*p* = 0.139). On d1, d15 and d30, the sexes differ significantly, with *p*-values ranging from 0.002 to 0.014. In (**b**), the differences in protective PP in the serum are shown. There are no significant differences between the sexes in baseline (MHAC and FHAC) and MOG-immunized animals (MMOG and FMOG). Except for d1, there is a significant difference detectable over all time points between the sexes, ranging from *p* = 0.001 to *p* = 0.046.

Figure 8 shows the results of TAC and PP. A significant difference was not detectable between the male and female animals amongst healthy appearing controls (MHAC vs. FHAC). On d1 after cytokine injection, females showed a strikingly increased TAC compared to the male counterparts (*p* = 0.014). On d3, however, a detectable difference between the sexes was not observed after a substantial decrease in TAC in females to the levels comparable to those observed in males. On d15 and d30, females exhibited strong elevations in TAC, contrary to the rather stable TAC in males, resulting in statistically significant differences between the two sexes (d15, *p* = 0.002; d30, *p* = 0.005). A detectable increase in TAC in the male group was observed at d45*, reaching to the levels detected in females. PP concentrations in the sera of female rats showed significant increases between d3 and d45* (*p* = 0.001–0.040) in spite of the balanced baseline values between both sexes (MHAC 10.90 [95% CI = 9.89–11.40] mM and FHAC 10.90 [95% CI = 10.60–11.10] mM).

## 4. Discussion

Overall, our data suggest a positive effect of VD supplementation in maintaining cortical structures and regulating oxidative stress in both sexes at all time points in our animal model of MS. There was a significantly better preservation of myelin (PLP) structures in males on d30 and in females on d15. Whilst significant differences in males were only detected on the cerebral cortices ipsilateral to the catheter implantation, females showed significant differences on the contralateral brain hemisphere. Our animal model did not show significant difference in PLP loss detectable between males and females on d30 or d45*. Nevertheless, males had significantly more PLP loss on d15 in both hemispheres than females, indicating a faster progression of the disease phenotype, reminiscent of the sex differences observed in human patients with MS.

Besides the fact that males have a different composition of CSF serum-derived proteins than females, CSF flow dynamics are also sex-dependent [18,19]. Presumably, this is the case in our model too, where the proinflammatory cytokines were given at a speed comparable to that of the physiological CSF flow. There might be a different CSF flow dynamic in female rats than in male rats, resulting in a different pattern of cortical pathology. This probably also explains the shift in the disease peak between the sexes from d15 in males to d30 in females.

Another explanation could be the protective effect of estrogens as reported in previous studies [20], leading to a delay in the disease peak. Whilst VD has been presumed to enhance remyelination [21], in this study, it most likely also affects myelin preservation. Some studies point toward a higher myelin repair efficacy in female rodents [2]. Our model did not reveal any significant differences in PLP structures between the sexes on d30 or d45*, where remyelination is detectable. Because of the different peaks of the disease and the delay in female rats, this difference in remyelination capacity might only be detectable in a more extended experiment than in our current setup that spans a maximum of a 45-day period.

Studies on pathology and magnetic resonance imaging in MS patients have shown that the higher neurological progression observed in males was associated with axonal loss, grey matter and entire brain atrophy. Research on both human and animal models of multiple sclerosis indicates that pro-inflammatory T cells, increased activation of astrocytes, and iron release from oligodendrocytes may cause male-aged microglia at the edge of white matter lesions to become more reactive [2]. In our study, microglial activation was significantly lower on d30 and d45* on both hemispheres in VD^+^ male animals. In contrast, females showed significantly lower microglial activation on d15 and d30 both sides, pointing towards a delayed pathological progression in females. In particular, a protective effect of estrogens on microglia is described in the literature [20] and might explain the mitigated microglial activation in females compared to males on d45*. Microglial inflammation may be an indicator of axonal injury, which exacerbates neuronal dysfunction. Since activated microglia contribute to demyelination, possibly due to altered production of nitric oxide and cytokines, axonal injury and thus neuronal dysfunction is caused [7]. Our data support a role for estrogen in the attenuation of microglia-mediated neuronal injury in the preclinical PMS phenotype, although obtaining conclusive evidence for a causal correlation requires additional experiments.

A potent effect of VD supplementation in all animal groups could be observed on apoptotic cells, with a significantly lower apoptotic cell count in all VD^+^ groups except for Fd45*. VD regulates cell proliferation and apoptosis within the rat cortex in the developing brain at both the cellular and molecular levels [22]. VD also seems to have neuroprotective effects on hippocampal apoptosis in rats [23]. In line with these reports, our results support a protective effect of VD on apoptotic cell death in the adult rat cortex. Interestingly, there are significantly more apoptotic cells in females on d15 detectable compared to the male counterparts. This observation seems to contradict the better preservation of all other cortical structures in females. It should be noted that our previous work showed that apoptotic cells were mostly astrocytes [12]. Female animals may have more reactive astrocytes, and these cells could become damaged and degenerate because of acidosis, oxidative stress, substrate deprivation or certain cytokines, while helping to alleviate the detrimental outcome. This could further explain the difference between the sexes and the delayed damage in females, where the astrocytic reaction might start earlier and perhaps be more potent, tempering the cortical pathology.

VD supplementation leads to significantly greater neuronal survival in male VD^+^ animals at d15 and d30 as well as in females at d15. Since there is an increased loss of neurons detectable in untreated males at d15, this sex benefits even more from VD supplementation compared with females with respect to neuronal preservation. Male neurons are more susceptible to oxidative stress, possibly related to sex differences in the mitochondrial respiratory system and antioxidant defense mechanisms, and sex chromosomes seem to complement and contribute to these phenotype differences [2]. Consistent with these reports, our results demonstrated a significantly greater loss of neurons and a weaker oxidative stress defense. Concerning the TAC in males compared to females, no significant difference is detectable in healthy-appearing controls (HAC). However, in the experimental groups, female animals exhibited significantly higher TAC than male rats at d1, d15 and d30, indicating that TAC plays a much more critical role in disease prognosis, whilst it does not necessarily render females less susceptible to disease onset.

Additionally, females also had higher protective PP concentrations in their sera detectable from d3 onwards. Given that the sex hormones are known to influence the immune system differentially, the better female immune response could be attributed to estrogens [3]. Also, the iron load is higher in men in the deep grey matter regions, which may contribute to greater oxidative damage and inflammation [2]. Oxidative stress is suggested to contribute to demyelination and axonal damage in both MS and animal models [8]. Proposed mechanisms comprise mitochondrial injury caused by oxidative stress, oxidative burst in microglia and oxidized lipids [8,24]. Oxidative stress-mediated pathways and cellular effects are involved in immune cell priming in the peripheral lymphoid organs. Research on certain pathways and cell interactions in appropriate models could aid in understanding processes regulating the formation and progression of the lesions [16], as the oxidative brain environment is altered in MS patients [8]. We investigated the presence of two different oxidized lipids in our animal groups in histology. While VD has a significant protective effect in all male groups, it demonstrated significant protection in females only at d30 (HOCL-oxLDL) and d45* (Cu++-oxLDL). Despite these divergent effects, significant differences between the two sexes could only be observed on d30 in HOCL-oxLDL levels. Given the delay in developing cortical pathology in females, it appears feasible to presume a concordant delay in developing oxidative-stress epitopes, explaining the different patterns of histological stress markers between the two sexes. In general, males are believed to experience more oxidative stress than females, which might explain the higher effect of immunoregulatory effects of VD on males [25]. While the courses of HOCL-oxLDL and Cu++-oxLDL were similar in males, the two markers exhibited slightly diverse patterns in female rats. HOCl is produced by myeloperoxidases, mainly affecting proteins, while Cu++ oxidation involves non-enzymatic pathways, mainly affecting lipids [26,27]. Those differences can even effect and influence apoptotic mechanisms by activating different apoptotic cell programs [28]. Because of the observed differences in apoptotic cell counts in the present study, a closer analysis of these pathways would provide interesting insights.

In humans, there is no significant difference in sNfL levels detectable between male and female PMS patients. NfL in general reflects acute disease activity and axonal loss in MS patients at high risk of progression [29]. Previous studies show that serum NfL levels peak much earlier in male rats (d3) than the histological peak of cortical pathology is detectable (d15 for males). Since there is a significant increase in sNfL in female rats from d30 to d45 (after a second cytokine injection), it would be interesting to investigate how the histological features might emerge at later time points after d45* (Supplementary Table S3). Because the peak of the disease, determined using histological markers, is delayed in females after the first cytokine injection (d30 instead of d15), there might also be a delay in the second disease phase after the second cytokine injection on d30. To address this question, experimental timespan and blood sampling times will be adjusted accordingly in future studies. 

While VD supplementation leads to significant sNfL reduction in serum in males at d15, this effect is not detectable in the female group. Since NfL rises upon neuroaxonal damage, VD supplementation preserved neuroaxonal structures in male rats, although it did not entirely suppress the pathology. Because of the significant difference in cortical pathology between sexes at d15 with a higher PLP preservation in males, the overall reduced damage in females revealed lower sNfL values on d15, also underlining the effect of VD. It would be interesting to assess sNfL levels at d45* in VD^+^ and VD^−^ females in further studies.

In MS, systemic glucocorticoids assist with treating acute relapses but have no effect on long-term consequences. Cholesterol metabolites are the source of sex hormones, corticosteroids, and VD. They are nuclear receptor ligands, and as such, their activation controls the transcription of several genes involved in immunomodulation. Some influence the activity of myeloid and lymphoid cells, hence inducing a state of reduced inflammation. Smaller studies using testosterone have shown a neuroprotective effect, potentially in conjunction with increases in brain-derived neurotrophic factors and growth factors. In relapsing–remitting MS patients, testosterone has also been linked to a notable increase in grey matter in the frontal cortex. It can stop or reverse the neurodegenerative alterations related to MS. In the clinical care of MS, the use of sex steroids, either alone or in combination with other medications, is still under investigation because to the demand for therapeutic drugs that induce remyelination in MS patients [7].

Most animal studies have been conducted in a single sex under the assumption that the findings would hold true for both sexes. EAE studies especially show a clear female bias, with 85% of these studies only conducted on female rodents. Meanwhile, there is a clear sex difference in immune response, prevalence and progression of many diseases known, which makes further studies in both sexes, especially for the progressive disease phase, necessary [1,30,31]. Additionally, there is already some evidence from EAE models that some MS therapeutics may also have distinct effects in males and females. However, evidence for this phenomenon from MS patients is still lacking and many studies on the responses to disease-modifying therapies in MS patients did not stratify the data based on sex [1,32]. The fact that sexes differ in oxidative/antioxidative and inflammatory stress mechanisms underlines the importance of considering sexual dimorphism [25].

VD plays an essential part in immune system modulation, which impacts health maintenance and disease development. Although a low VD serum level is associated with an increased risk of autoimmune disease onset and higher disease activity, the most beneficial times and amounts of supplementation are not well defined since they can differ between patients and are dependent on factors like age and sex. Hypovitaminosis D is generally more common in the elderly, and blood levels of VD appear to be influenced by sex. From a therapeutic perspective, more research is needed to answer these questions because VD supplementation may be a useful tool for the additional treatment of autoimmune diseases when it is tailored to a patient’s age and gender [5].

In 2020, the recommendation of chronic supplementation in adults was to not exceed 600 IU/day, while at the same time presenting data that even 10,000–40,000 IU/day appeared safe as an add-on therapy in autoimmune diseases [33]. One recent recommendation for VD supplementation for all healthy adults in Europe is approximately 1000 IU daily. This would raise the VD serum levels in 95% of the population to >50 nmol/L [34]. In another systematic review, data confirmed that keeping the VD serum concentrations above 50 ng/mL (125 nmol/L) reduces the risk from community outbreaks, sepsis and autoimmune disorders [35]. These differences in dosage recommendations highlight the need among both healthy and diseased human beings for monitoring VD serum levels and correspondingly adjusting VD dosage. Only when reaching an individual proper dose of VD can effectiveness be ensured and any adverse effects due to overdosing be avoided [34]. Besides finding the correct dosage of VD, the mode of supplementation also plays a critical role. While intermittent doses at intervals longer than once a month are unphysiological and thus ineffective, studies have shown that daily VD supplements are more beneficial than less frequent supplementation [35].

There is evidence for a higher protective effect of VD-based therapeutic approaches in women, at least at fertile age, than in men, likely due to the crosstalk between estrogen and VD [5]. Therefore, a synergy for immunomodulatory effects of VD and 17-beta-estradiol is discussed as a therapy option for relapsing–remitting MS patients [7]. However, in our study, male animals benefited even more from VD supplementation via boosting antioxidative capacity in males due to its antioxidative effects.

## 5. Conclusions

At least in our animal model, both male and female rats derive benefits from VD sup-plementation, represented by significantly less cortical, neuroaxonal and oxidative damage. Unexpectedly, male rats had an even higher overall benefit, most likely due to differences in oxidative capacity and defense systems. We extrapolate that these find-ings should further be investigated in clinical trials to learn more about these mecha-nisms in patients with MS.

## Figures and Tables

**Table 1:** Detailed overview of animals, tissue and serum samples used for the respective subgroups. In total, 96 DA rats were used, including 46 females and 50 males. Tissue samples were harvested on d15, d30 and d45*. Blood withdrawal was additionally performed 4 weeks after MOG immunization (FMOG, MMOG) and on d1 and 3. Abbreviations: female (F); male (M); healthy appearing control (HAC); vitamin D (VD); myelin oligodendrocyte glycoprotein (MOG); day (d); immunohistochemistry (IHC); total antioxidative capacity (TAC); polyphenols (PP); neurofilament light chain (NfL); single-molecule array (SIMOA); not determined (n.d.); supplementary material (s.m.). The asterisk at d45 indicates the second cytokine injection on d30.

	Sacrifice	Number of Serum Samples		
	Tissue for IHC *n* =	Used for TAC *n* =	Used for PP *n* =	Used for NfL SIMOA *n* =
**FHAC**	7	5	14	5
**FMOG**	No sacrifice	5	10	n.d.
**Fd1**	No sacrifice	4	9	n.d.
**Fd1VD**	No sacrifice	n.d.	n.d.	n.d.
**Fd3**	No sacrifice	4	10	n.d.
**Fd3VD**	No sacrifice	n.d.	n.d.	n.d.
**Fd15**	7	5	10	5
**Fd15VD**	6	n.d.	n.d.	7
**Fd30**	7	4	10	s.m.
**Fd30VD**	6	n.d.	n.d.	n.d.
Fd45 *	8	5	10	s.m.
Fd45 *VD	5	n.d.	n.d.	n.d.
**MHAC**	9	8	16	9
**MMOG**	No sacrifice	11	20	n.d.
**Md1**	No sacrifice	8	19	n.d.
**Md1VD**	No sacrifice	n.d.	n.d.	n.d.
**Md3**	No sacrifice	7	12	n.d.
**Md3VD**	No sacrifice	n.d.	n.d.	n.d.
**Md15**	10	10	14	9
**Md15VD**	8	n.d.	n.d.	9
**Md30**	5	10	14	n.d.
**Md30VD**	7	n.d.	n.d.	n.d.
Md45 *	4	5	7	n.d.
Md45 *VD	7	n.d.	n.d.	n.d.
**TOTAL FEMALE**	**46**	**32**	**73**	**17**
**TOTAL MALE**	**50**	**59**	**102**	**27**
**TOTAL n =**	**96**	**91**	**175**	**44**

**Table 2:** Statistical analysis of histological quantification. Most of the groups were not normally distributed after visual inspection of the histogram and Q–Q plots as well as after the Shapiro–Wilk test. The Kruskal–Wallis test showed that there are significant detectable differences between the groups. Pairwise analysis using the Mann–Whitney U test revealed significant differences in the following groups as listed in the table below. A difference of *p* < 0.05 was considered to be statistically significant (bold and underlined). The asterisk at d45 indicates the second cytokine injection on d30.

	PLP		Iba1		Casp3	NeuN	Cu++ oxLDL	HOCL oxLDL
	Ipsi	Contra	Ipsi	Contra	Ipsi	Ipsi	Ipsi	Ipsi
**Shapiro–Wilk, Histogram, Q-Q-Plot**	Most of the groups are not normally distributed							
**Kruskal–Wallis test *p***	<0.001	<0.001	<0.001	<0.001	<0.001	<0.005	<0.001	<0.001
**Md15 vs. Md15VD**	0.142	0.064	0.197	0.197	**0.002**	**0.019**	**0.002**	**0.001**
**Md30 vs. Md30VD**	**0.050**	0.122	**0.004**	0.123	**0.004**	**0.019**	**0.004**	**0.042**
Md45 * vs. Md45 *VD	0.298	0.705	**0.018**	**0.038**	**0.023**	0.186	**0.011**	**0.010**
**Fd15 vs. Fd15VD**	0.122	**0.027**	0.109	**0.005**	**0.003**	**0.022**	0.108	0.688
**Fd30 vs. Fd30VD**	0.229	0.116	**0.025**	**0.004**	**0.046**	0.199	0.617	**0.004**
Fd45 * vs. Fd45 * VD	0.605	0.941	0.144	0.100	0.123	0.883	**0.042**	0.242
**Md15 vs. Fd15**	**0.022**	**0.003**	0.828	0.828	**0.001**	**0.042**	0.335	0.199
**Md30 vs. Fd30**	0.251	0.249	0.143	0.583	0.062	0.808	1.000	**0.006**
Md45 * vs. Fd45 *	0.496	1.000	**0.050**	**0.010**	0.186	0.496	0.450	0.396

**Table 3:** Statistical analysis of serum markers. Most of the groups were revealed not to be normally distributed after checking the histogram and Q–Q plots in addition to the Shapiro–Wilk test. The Kruskal- Wallis test showed that there are significant detectable differences between the groups. The Mann–Whitney U test revealed significant differences in the following groups, compared pair-wise and listed in the table below (bold and underlined). A difference of *p* < 0.05 was considered to be statistically significant. The asterisk at d45 indicates the second cytokine injection on d30.

	TAC	PP	NfL
**Shapiro–Wilk, Histogram, Q-Q-Plot**	Most of the groups are not normally distributed		
**Kruskal–Wallis test *p***	<0.001	<0.001	<0.001
**MHAC vs. FHAC**	0.306	0.454	**0.016**
**MMOG vs. FMOG**	0.139	0.180	x
**Md1 vs. Fd1**	**0.014**	0.065	x
**Md3 vs. Fd3**	0.850	**0.001**	x
**Md15 vs. Fd15**	**0.002**	**0.001**	**0.004**
**Md30 vs. Fd30**	**0.005**	**0.046**	x
Md45 * vs. Fd45 *	0.754	**0.040**	x
**Md15 vs. Md15VD**	x	x	**0.006**
**Fd15 vs. Fd15VD**	x	x	0.683

## Data Availability

All original material and data are saved at the Department of Neurology, Medical University of Graz. The data presented in this study are available upon request from the corresponding author.

## Supplementary Materials

The following supporting information can be downloaded at: https://www.mdpi.com/article/10.3390/nu16040554/s1, Table S1: A detailed list of all used important abbreviations.; Table S2: Primary and secondary antibodies used during this study with detailed information.; Table S3: sNfL results of female VD- rats.
