# Supplementary material for: Sex Differences under Vitamin D Supplementation in an Animal Model of Progressive Multiple Sclerosis
Source: Nutrients. 2024 Feb 17;16(4):554. doi: 10.3390/nu16040554 (PMC10893160; doi:10.3390/nu16040554)
Supplement: Supplementary file 1 [file nutrients-16-00554-s001.zip › nutrients-2846412-supplementary.pdf]

Supplementary Table S1 A detailed list of all used important abbreviations.

Group names	**FHAC**	Female healthy appearing control
	**FMOG**	Female myelin oligodendrocyte glycoprotein immunized rats
	**Fd1**	Female rats, day 1 after cytokine injection
	**Fd1VD**	Female rats, day 1 after cytokine injection, vitamin D supplemented
	**Fd3**	Female rats, day 3 after cytokine injection
	**Fd3VD**	Female rats, day 3 after cytokine injection, vitamin D supplemented
	**Fd15**	Female rats, day 15 after cytokine injection
	**Fd15VD**	Female rats, day 15 after cytokine injection, vitamin D supplemented
	**Fd30**	Female rats, day 30 after cytokine injection
	**Fd30VD**	Female rats, day 30 after cytokine injection, vitamin D supplemented
	**Fd45***	Female rats, day 45 after two cytokine injections (one on day 0, one on day 30)
	**Fd45*VD**	Female rats, day 45 after two cytokine injections (one on day 0, one on day 30), vitamin D supplemented
	**MHAC**	Male healthy appearing control
	**MMOG**	Male myelin oligodendrocyte glycoprotein immunized rats
	**Md1**	Male rats, day 1 after cytokine injection
	**Md1VD**	Male rats, day 1 after cytokine injection, vitamin D supplemented
	**Md3**	Male rats, day 3 after cytokine injection
	**Md3VD**	Male rats, day 3 after cytokine injection, vitamin D supplemented
	**Md15**	Male rats, day 15 after cytokine injection
	**Md15VD**	Male rats, day 15 after cytokine injection, vitamin D supplemented
	**Md30**	Male rats, day 30 after cytokine injection
	**Md30VD**	Male rats, day 30 after cytokine injection, vitamin D supplemented
	**Md45***	Male rats, day 45 after two cytokine injections (one on day 0, one on day 30)
	**Md45*VD**	Male rats, day 45 after two cytokine injections (one on day 0, one on day 30), vitamin D supplemented
Tissue markers	**PLP**	Proteolipid protein, myelin protein marker
	**Iba1**	Ionized calcium-binding adaptor molecule 1, marker for activated microglia

	**Casp3**	Caspase 3, marker for apoptotic cells
	**NeuN**	Neuronal-Nuclei, marker to detect most postmitotic neuronal cell types
	**Cu++oxLDL**	Cu^{++} oxidized low-density lipoprotein
	**HOCLoxLDL**	Hypochlorous acid oxidized low-density lipoprotein
**Serum markers**	**TAC**	Total anti-oxidative capacity
	**PP**	Polyphenols
	**sNfL**	Serum neurofilament light chain

Supplementary Table S2 Primary and secondary antibodies used during this study with detailed information.

**Antibody**	**Target**	**Host**	**Dilution**	**Marker/ Purpose**	**Company and Catalog number**	**AB ID**
Caspase3	Anti-active Caspase3 antibody	rabbit	1:500	Apoptosis	Abcam ab2302	AB_302962
Cu^{++}oxLDL	Anti-lipoprotein (a), oxidized via Cu^{++}	sheep	1:3000	Oxidative Stress	FT-OL11	
GFAP	anti-GFAP Ab-6 (Clone ASTRO6)	mouse	1:100	Activated Astrocytes	Thermo Scientific MS-1376	AB_1095984
HOCloxLDL	Anti-modified LDL (via HOCl)	rabbit	1:2000	Oxidative Stress	HL02	
Iba1	Anti Ionized calcium-binding adaptor molecule 1	rabbit	1:1000	Mikroglia	Wako 019-19741	AB_839504
NeuN	Anti- Neuronal-Nuclei, clone A60 (KC)	mouse	1:100	Neurons	Millipore MAB377-KC	AB_2298772
NfH	Anti-200kD Neurofilament Heavy Chain antibody	rabbit	1:2000	Neuro-filament	Abcam ab8135	AB_306298
PLP	Anti-myelin	mouse	1:500	De-	AbD	AB_22371

	proteolipid protein			myelination	Serotec<br>MCA839 G	98
anti-rabbit ImmPRESS reagent	anti-rabbit IgG	horse	ready to use	Secondary antibody	Vector<br>MP-7401	AB_2336529
anti-mouse ImmPRESS reagent	anti-mouse IgG	horse	ready to use	Secondary antibody	Vector<br>MP-7422	AB_2336527

Supplementary Table S3 sNfL results of female VD$^-$ rats.

	FHAC	Fd15	Fd30	Fd45*
N =	5	7	4	5
Median	5.11	5.90	9.10	37.00
Interquartile range	1.83	4.15	14.03	46.65
VS Fd15 p =	0.295	x	0.086	**<u>0.009</u>**
VS Fd30 p =	**<u>0.027</u>**	0.086	x	**<u>0.027</u>**
VS Fd45* p=	**<u>0.009</u>**	**<u>0.009</u>**	**<u>0.027</u>**	x
